# Mechanical Properties of Different Nanopatterned TiO_2_ Substrates and Their Effect on Hydrothermally Synthesized Bioactive Hydroxyapatite Coatings

**DOI:** 10.3390/ma13225290

**Published:** 2020-11-23

**Authors:** Amanda Bartkowiak, Arkadiusz Zarzycki, Slawomir Kac, Marcin Perzanowski, Marta Marszalek

**Affiliations:** 1 Institute of Nuclear Physics PAN, Radzikowskiego 152, PL-31342 Krakow, Poland; arkadiusz.zarzycki@ifj.edu.pl (A.Z.); marcin.perzanowski@ifj.edu.pl (M.P.); marta.marszalek@ifj.edu.pl (M.M.); 2 Faculty of Metals Engineering and Industrial Computer Science, AGH University of Science and Technology, Mickiewicza 30, PL-30059 Krakow, Poland; slawomir.kac@agh.edu.pl

**Keywords:** hydroxyapatite coating, nanotubes, scratch test, bioactive coatings, anodized titanium

## Abstract

Nanotechnology is a very attractive tool for tailoring the surface of an orthopedic implant to optimize its interaction with the biological environment. Nanostructured interfaces are promising, especially for orthopedic applications. They can not only improve osseointegration between the implant and the living bone but also may be used as drug delivery platforms. The nanoporous structure can be used as a drug carrier to the surrounding tissue, with the intention to accelerate tissue–implant integration as well as to reduce and treat bacterial infections occurring after implantation. Titanium oxide nanotubes are promising for such applications; however, their brittle nature could be a significantly limiting factor. In this work, we modified the topography of commercially used titanium foil by the anodization process and hydrothermal treatment. As a result, we obtained a crystalline nanoporous u-shaped structure (US) of anodized titanium oxide with improved resistance to scratch compared to TiO_2_ nanotubes. The US titanium substrate was successfully modified with hydroxyapatite coating and investigated for bioactivity. Results showed high bioactivity in simulated body fluid (SBF) after two weeks of incubation.

## 1. Introduction

The search for a suitable material that can replace or repair bone defects and at the same time prevent postoperative infections has been of great interest in the field of biomaterials and tissue engineering. New materials should follow strict biological and mechanical requirements in order to be applied as medical devices. Lately, the improvement of orthopedic implants has spurred the research toward surface design, especially in relation to the synergic effects of the following features: bioactivity, osteoconductivity, and antibacterial properties [1,2]. Among different approaches to tailor and functionalize surfaces, nanotechnology offers promising methods to optimize the surface of biomaterials on a nanoscale level.

Titanium and its alloys are widely used as intra-osseous implants [3]. Titanium implants drive their resistance to corrosion and the ability to integrate with bone from a stable oxide film that is formed on their surface [4,5]. Clinical tests show that not only surface chemistry exerts an influence on osseointegration mechanisms but also surface roughness. The smooth and untreated titanium surface exhibits poor fixation with bone tissue. After implantation, even highly biocompatible metals are separated from bone by a thin layer of soft tissue, called fibrosis, that prevents the implant surface from being in direct contact with bone [6,7,8]. Nevertheless, fibrotic encapsulation is a natural outcome of the healing process; it walls off the implant from bone tissue. Desirable healing response to a bioactive surface just after the inflammation stage leads to the vascularization and formation of fibrocartilage and osteoid, which is subsequently followed by bone maturation [9]. This type of body response is highly recommended for orthopedic applications, as it provides rigid fixation at the bone–implant interface.

Nanoscale structures offer surface energy larger than other texture scales, which can improve the adhesion of proteins present in the extracellular matrix (fibronectin, vinculin). The immobilization of biomolecules, such as enzymes or matrix proteins, can be used to stimulate cellular adhesion and migration, which is known as a substantial step in osseointegration processes [10,11]. Functionalizing a surface with other bioactive molecule, such as lectins, can exert different effects on the cellular level, such as the immunomodulatory effect (stimulating the proliferation of lymphocytes and splenocytes) or the inhibition of bacterial and fungal growth [12,13].

The electrochemical anodization of titanium gives the opportunity to produce nanotubular or nanoporous surface structures, yielding bioactive and osteoconductive properties [14,15]. Studies show that such a specific crystalline nanotubular structure of TiO_2_ significantly increases new bone formation in vivo and gene expression associated with osteogenesis, and it also enhances osteoblasts proliferation and adhesion, as well as generates a high activity of alkaline phosphatase. In comparison, smooth or micro-rough surfaces were shown to have an insignificant effect on osteogenesis mechanisms [16,17].

Another important aspect of nanotubes or nanopores is their promising application as smart drug delivery platforms [18,19]. The localized controlled release of therapeutics is a forward-looking strategy for inflammatory and antibacterial treatment after implantation. TiO_2_ nanotubes (NT) have been shown to exhibit great potential for such applications [20]. Despite these attractive physicochemical and biological properties of TiO_2_ NT, their main limitation is poor mechanical performance. Subjected to compression loads, NT were shown to experience brittle fracture with fragmentation [21].

In this work, we demonstrate a crystalline nanoporous u-shaped structure (US) of anodized titanium that revealed twice higher resistance to scratch compared to NT. The TiO_2_ US substrate could be used as a drug carrier to the surrounding tissue or as a surface with nanoroughness suitable for the immobilization of biomolecules. Moreover, the US substrate was successfully functionalized with hydroxyapatite coating (HApUS) and investigated for bioactivity. The combination of such specific nanotopography and bioactivity of HAp was previously reported to have a positive synergic effect on osseointegration in vivo [22]. Specifically, it was shown to upregulate the gene expressions of cell adhesion and osteogenic differentiation markers. We are convinced that the HApUS composite could be an upgrade of the implant surface-stimulating osteogenesis mechanisms while maintaining mechanical stability during surgery.

## 2. Materials and Methods

### 2.1. Preparation of Titanium Substrates

The preparation of anodized titanium substrates was carried out according to the study of Suchanek et al. [23]. Prior to the anodization process, pure titanium foil (BIMO Metals, Wroclaw, Poland) with a thickness of 3 mm was cut into square pieces of 14 × 14 mm^2^ and subsequently polished to mirror quality with CeO_2_ paste for noble metals (Surex, Poland). Afterwards, samples were cleaned chemically by immersion in an aqueous acid mixture of 5.6 M HNO_3_ and 3.3 M HF (POCH, Poland) for 2 min. This treatment was followed by washing with distilled water and ultrasonic cleaning in ethanol for 5 min.

**Figure 1:**
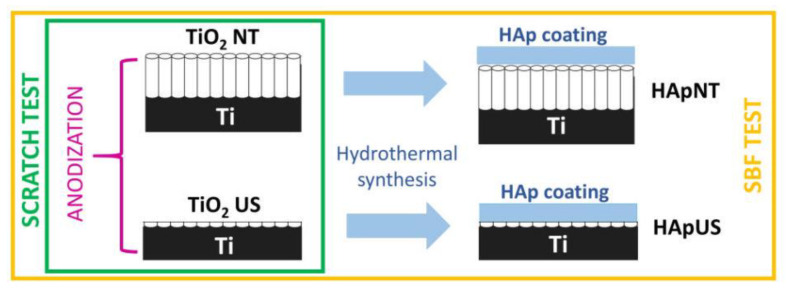
Schematic overview of sample preparation, including the formation of TiO_2_ layers by an anodization process (u-shaped structure (US) and nanotubes (NT)) and a hydrothermal synthesis of hydroxyapatite (Hap) coating on thermally treated nanopatterned substrates (HApNT and HApUS).

Fabrication of the TiO_2_ layer in the form of nanotubes and u-shaped nanopits was carried out on a Teflon holder by standard two-electrode anodization in potentiostatic mode at room temperature. During this process, voltage was kept constant at 50 V, Ti substrate was used as an anode, and a platinum plate was used as a cathode. The electrolyte used in the anodization process of both NT and US contained 0.5 wt % NH_4_F (POCH, Poland) and 1 wt % H_2_O dissolved in ethylene glycol. Samples were prepared with a two-step anodization process, where after the first anodization performed for 120 min, the oxidized layer was removed ultrasonically. This step was followed up by a second anodization process, when nanotubes were anodized for another 120 min, and specimens with u-shapes were anodized for 15 s. In order to wash out the remains of the electrolyte, samples were cleaned for 10 min in an ultrasonic bath with ethanol. Afterwards, both kinds of nanostructured samples, NT and US, were annealed in an oxidizing atmosphere at 600 °C for 1 h. Taking into account that US are produced as a result of NT removal, followed by a short anodization process, the US can be considered as an initial stage of NT formation. The schematic overview of sample preparation is demonstrated in Figure 1.

### 2.2. Hydrothermal Synthesis of Hydroxyapatite Coatings

**Figure 2:**
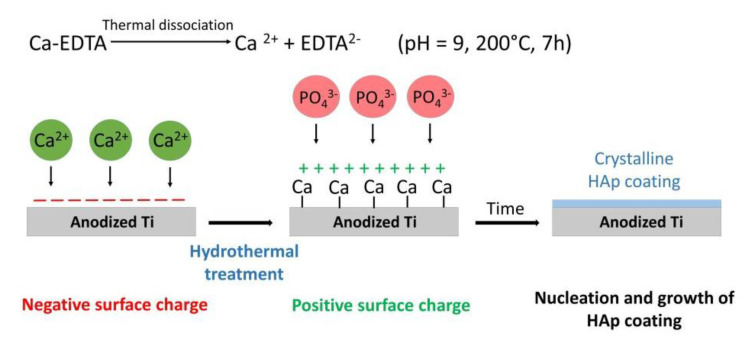
Schematic presentation of chemical reactions occurring during the hydrothermal synthesis of HAp coatings on anodized and annealed Ti substrates in the form of US and NT.

The synthesis of hydroxyapatite (HAp) coatings on substrates with a nanopatterned TiO_2_ intermediate layer (NT and US) was carried out under hydrothermal conditions according to the method described in our previous study [24]. An overview of the reactions occurring during the hydrothermal synthesis of HAp coatings is demonstrated in Figure 2. Firstly, an aqueous solution was prepared with a Ca/P ratio of 1.67 from the following ingredients: Ca(NO_3_)_2_·4H_2_O (0.2 M) (POCH, Gliwice, Poland), (NH_4_)_2_HPO_4_ (0.12 M) (POCH, Gliwice, Poland), and Na_2_EDTA·2H_2_O (0.2 M) (POCH, Gliwice, Poland). The pH of the calcium–phosphate solution was adjusted to 9.0 with a dropwise addition of ammonium hydroxide. Secondly, the nanopatterned substrates, one at a time, were fixed on a titanium holder and placed in a 200 mL glass container inside the autoclave. The holder was set at the angle of 45° to the bottom. Finally, the calcium–phosphate solution was poured into the glass vessel, allowing the samples to be completely immersed. The synthesis was carried out for 7 h at 200 °C in a sealed autoclave. After the hydrothermal process, samples were rinsed with distilled water and dried in air at room temperature.

### 2.3. Morphology, Structure, and Adhesion Characterization

The surface morphology and chemical characterization of samples was done using scanning electron microscopy (SEM, Tescan Vega 3, Fuveau, France), equipped with an energy-dispersive X-ray spectrometer (QUANTAX EDS, Bruker, Billerica, MA, USA). The chemical composition was examined using a Raman spectrometer (Almega XR of Thermo Electron Corp., Winsford, UK) equipped with an optical microscope. The Raman signal was excited at the wavelength of 532 nm. Data were collected in the spectral range from 100 to 4000 cm^−1^ with resolution of 2 cm^−1^. The XRD measurements were carried out with a PANalytical X’Pert Pro diffractometer (Almelo, Netherlands) in the standard θ–2θ geometry. The operating voltage and current were kept at 40 kV and 30 mA, respectively. The acquisition of XRD diffraction patterns was performed with a step size of 0.05° in the 2θ range from 20° to 100° using Cu Kα (λ = 1.54 Å) radiation. The angular resolution of the instrument was calibrated using an LaB6 line profile standard (SRM660a - NIST certificate [25]). We evaluated the adhesion strength of the nanopatterned samples using a combination of SEM, EDS, and a Nano-Scratch Test System (CSM Instruments, Needham, MA, USA). Scratch measurements were performed with normal load ranging from 0.1 to 100 mN and loading speed of 2 mN/s using a diamond Rockwell-tip with a top radius of 2 µm. On each type of the nanostructured titanium oxide film, we performed 10–15 scratches in different places of the samples. 

### 2.4. Bioactivity Test in SBF

A bioactivity assessment of US and NT samples, with and without HAp coating (HApUS, HApNT), was investigated by immersion in acellular simulated body fluid (SBF) according to ISO standard 23317:2014(E) [26]. The ionic concentration of SBF was nearly equal to that of human blood plasma at 36.5 °C (Na^+^ 142.0, K^+^ 5.0, Mg^2+^ 1.5, Ca^2+^ 2.5, Cl^−^ 147.8, HCO^3−^ 4.2, HPO_4_^2−^ 1.0, and SO_4_^2−^ 0.5 mM), and pH was adjusted to the value of 7.40 using TRIS and HCl (1 mol dm^−3^). Each of the samples was incubated for two weeks at 37 °C in plastic sterile containers with 7 mL of SBF. The fluid was refreshed every three days. After two weeks, samples were removed from SBF, rinsed with distilled water, and left to dry in air. SEM study was performed on as-prepared samples before and after immersion in SBF. The time of incubation was adjusted to observe full biomineralization in vitro.

## 3. Results

### 3.1. Morphology and Structure of Nanopatterned Crystalline TiO_2_ Layers before and after Hydrothermal Synthesis

**Figure 3:**
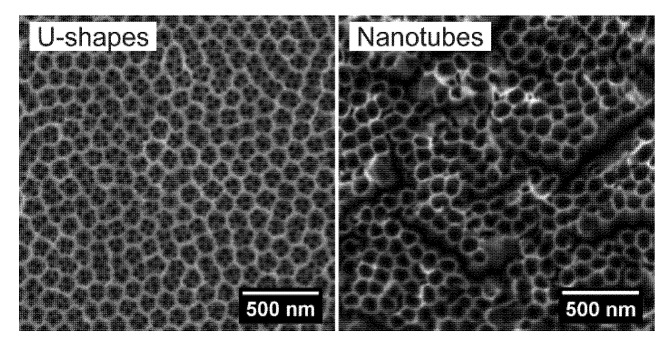
SEM images of anodized and annealed TiO_2_ layer in the form of US and NT.

SEM images presenting the morphology of annealed TiO_2_ u-shaped nanopits and aligned nanotubes are shown in Figure 3. Both forms of anodized TiO_2_ nanopatterns exhibit similar inner diameters of 106 ± 8 nm for US and 104 ± 10 nm for NT, which were determined directly from SEM pictures with the assumption of a circular shape of the structures. The height of the US is approximately ½ of its diameter, while in the case of NT, the 2 h long anodization process produces a micrometer long tubular structure. Moreover, we observed distinguishable small pores within the US arrays, with diameters of approximately 30 ± 4 nm.

**Figure 4:**
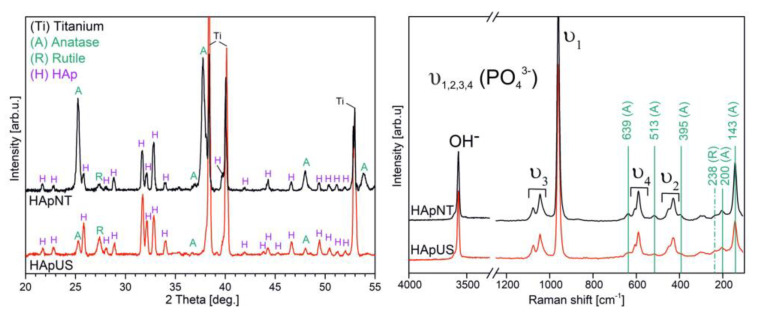
XRD patterns and Raman spectra of anodized and annealed TiO_2_ in the form of US and NT after the hydrothermal process (HApUS and HApNT, respectively).

Figure 4 demonstrates an XRD and Raman study on hydrothermally treated U shapes and nanotubes (HApUS and HApNT, respectively). From the structural and chemical point of view, the fabricated US can be considered as a similar product to NT composed of two crystalline forms of titanium oxide: anatase and rutile. The XRD patterns for HApUS and HApNT samples (Figure 4) showed Bragg peaks characteristic for anatase (according to the ICSD card No. 00-004-0477), rutile (according to the ICSD card No. 00-034-0180), and α-titanium (according to the ICSD card No. 01-089-5009). The XRD patterns of hydrothermally treated US and NT confirmed also the synthesis of the HAp phase with hexagonal symmetry and space group P63/m [24,27,28]. The lattice parameters calculated for the HAp coating on US (HApUS) were a = 9.43(1) Å and c = 6.89(1) Å, whereas for the HAp coating on NT (HApNT), they were a = 9.44(1) Å and c = 6.90(1) Å. These values agree with data established for the HAp phase [29,30]. Comparing the relative intensities of reflections for the HAp structure at 2θ of 31.7°, 32.1° and 32.8°, corresponding to (211), (112), and (300) crystallographic planes, we can notice some slight differences between samples HApUS and HApNT. We associate these discrepancies with the crystallographic texture or some structural defects. Preferred crystallographic orientation might also cause an absence of XRD reflection assigned to anatase at 2θ of 37.7°, 53.7° in case of the HApUS sample. The TiO_2_ coating that is not produced by an electrochemical anodization process, but for instance by chemical and thermal treatment is found in rutile but not the anatase structure [24,31,32]. In our previous study concerning the synthesis of HAp coating on a nanotubular surface of TiO_2_ [23], we observed the tendency for crystal growth along the (001) crystallographic direction. It is noteworthy that no other forms of crystalline calcium phosphates were observed in the XRD analysis.

Analysis on the structural and chemical nature of the produced samples complementary to XRD with the use of Raman spectroscopy is shown in Figure 4. The Raman vibrational spectra of both specimens HApUS and HApNT exhibit characteristic bands for hydroxyapatite structure, including a band originating from the hydroxyl group (OH^−^) and four internal modes associated with the presence of the phosphate group PO_4_^3−^ (v_1_, v_2_, v_3_, v_4_) [27,33,34]. In both cases, we also observe five characteristic bands for anatase, which are localized at 395, 513, 143, 200, and 639 cm^−1^ [35]. In this study, the only characteristic Raman-active band for rutile observed in spectra of anodized and annealed titanium is at the position of 238 cm^−1^. The other molecule vibrations of rutile phase at 610 and 446 cm^−1^ overlap with the stronger intensity vibration modes assigned to the phosphate group (v_2_ and v_4_) [36].

### 3.2. Resistance against Scratch of Crystalline TiO_2_ Layers in the Form of NT and US

A combination of three methods was applied—a nano-scratch test combined with EDS and SEM measurements—to provide a comparative study on the resistance against scratch for US and NT. The conventional method for assessing adhesion of a film to the substrate requires determining the critical normal load at which the film starts to delaminate from the substrate. This estimation is based on precise measurements of friction force (with constant increase of normal load) and micrographs of the scratched surfaces from optical microscope. Due to the nano-scaled size of the nanostructures, it was difficult to determine the critical normal loads directly from optical micrographs or changes in the acoustic emission sensor output. For that reason, additional morphological and elemental analysis was required to determine the moment of coating delamination and exposition of the metallic substrate.

**Figure 5:**
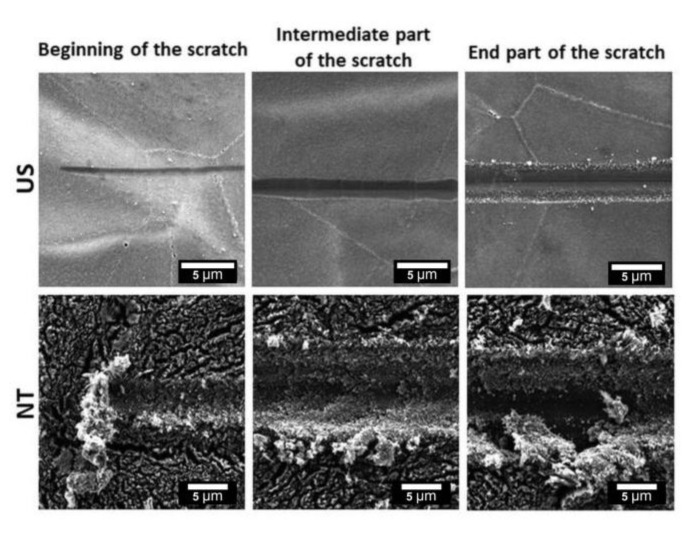
SEM images showing the evolution of grooves after a scratch test on samples with crystalline TiO_2_ layers in the form of US and NT.

Figure 5 presents SEM images of sample surfaces after the scratch test performed on different areas: the beginning of the scratch (left image); the intermediate area showing a deformed but still continuous (according to EDS measurement) layer of the crystalline TiO_2_ (middle image), and the area of layer delamination with substrate exposition (right image). From the SEM study, we observe that there is a significant difference in the deformation mechanism under increasing load applied on the moving tip between the US and sample with NT. Just after initial indentation at the minimal load force of 0.1 mN, nanotubes began to collapse, and many large debris along the scratch’s edges can be recognized. Further analysis along the groove confirmed nanotubes’ brittleness and ability to easily break and detach from the substrate. At the initial stage of the scratch test, we observe for the US sample superior mechanical properties, i.e., a shallow penetration of the Rockwell stylus and more plastic deformation compared to the NT layer. Further SEM analysis of the intermediate part of scratches on the US reveals substrate-like inner cracks within the groove caused by plastic deformation mechanisms. The presence of perpendicular cracks implies that the material underwent compaction and abrasion by the microploughing or microcutting mechanisms under the tip movement, although the US layer maintained its continuity and good adhesion. However, after reaching a certain depth of tip penetration, some debris can be recognized close to the groove’s edges presented in the delamination area (end part of the scratch), which may indicate the predominance of brittle deformation over plastic. In the failure point, also the morphology of the scratched US surface changed during tip sliding, and there was no evidence of nanopatterned features within the groove.

**Figure 6:**
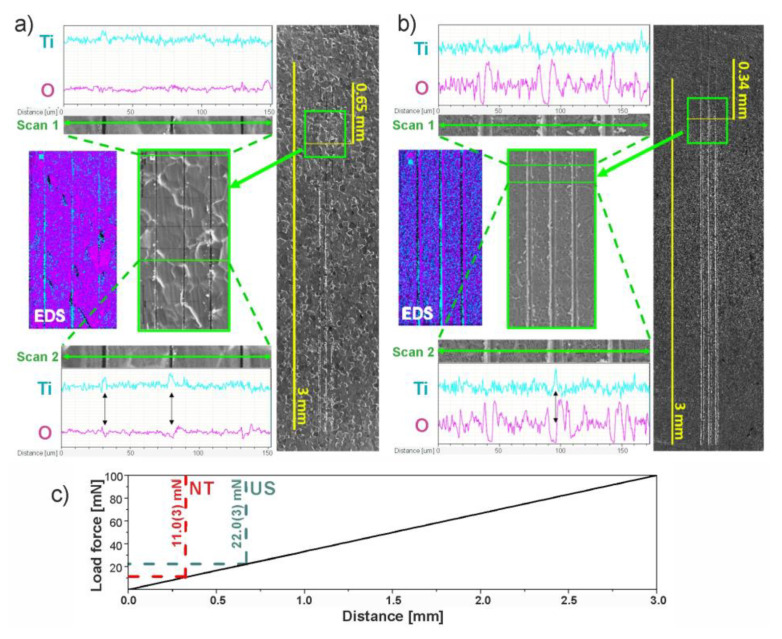
SEM and energy-dispersive X-ray spectrometer (EDS) analysis after a scratch test for (**a**) US and (**b**) NT: (on the right) SEM image of scratches where the numerical scale corresponds to the length of scratches (3 mm); (in the middle) high-magnification SEM image of a selected area marked as a green square; (on the left) EDS study of the selected area; (Scan 1 and 2) line profile EDS analysis across the scratches. Image (**c**) demonstrates dependence of the normal force on distance and estimated values of the critical normal force at which delamination of the thorough oxide film was observed for both the US and NT.

SEM images of selected areas where delamination of the TiO_2_ coating was identified after the scratch test, with their elemental mapping using EDS spectroscopy, are shown in Figure 6a,b for US and NT, respectively. Several profiles of EDS scans were made perpendicularly to the tip movement across all of the three grooves. Two representative profile spectra were selected to demonstrate data with titanium and oxygen signals, one showing the presence of a continuous coating within the grooves (Scan 1), and a second where delamination and exposure of the metallic substrate was found (marked with black arrows, Scan 2). The critical load was determined according to the distance at which EDS analysis showed a significant increase in signal for titanium and simultaneous decrease in signal for oxygen (Scan 2) at least for one of the grooves. In this work, the critical normal load at which we observed delamination of TiO_2_ film for the US sample was about twice higher than that for NT, i.e., the critical normal load was 22.0(3) mN for US and 11.0(3) mN for NT. The visible lines placed perpendicularly to the grooves on the SEM image of the US in Figure 6a are apparently caused by surface charging or superficial destruction of the TiO_2_ layer by electron beam during each EDS scan.

Scratches that are demonstrated in Figure 5 and Figure 6 are representative for each kind of TiO_2_ film. Figure 5 shows three sections (beginning/intermediate/end), where we demonstrate sequential changes in surface morphology along one scratch and differences in deformation mechanisms that correlate with results from SEM-EDS study in Figure 6. The beginning is the starting point of each scratch, the intermediate images were taken at a similar distance from the starting point, but the end images were made at different locations from the beginning of the scratch due to a significant difference in distance between the two samples NT and US where the delamination was observed.

The reason for the better mechanical performance of the US layer is strongly related to the aspect ratio between the walls height and diameter. As we outlined earlier, the height of the US array was approximately ½ of its diameter, while NT showed a micrometer-long tubular structure. Due to the ratio between the wall height and tube diameter, NT are prone to easily brake and collapse, which have been reported by other authors on the basis of nanoindentation studies [21,37]. When the penetration of an indenter proceeds in depth, NT fracture and interact with neighboring nanotubes, causing them to bend and fracture. In the last stage of this brittle deformation, we observe a densification of NT fragments (Figure 5).

### 3.3. Bioactivity Analysis

One of the most important requirements for modern biomaterials used in orthopedics is the formation of chemical bonding between the material’s surface and the living bone (osseointegration). This specific implant–tissue interaction should guarantee the proper stabilization of an implant inside the body and its long-term functionality. A common method for anticipating bone-bonding ability in vitro is by soaking a sample in SBF solution. After a certain period of immersion, the formation of bone-like apatite on the material’s surface should be observed [38].

**Figure 7:**
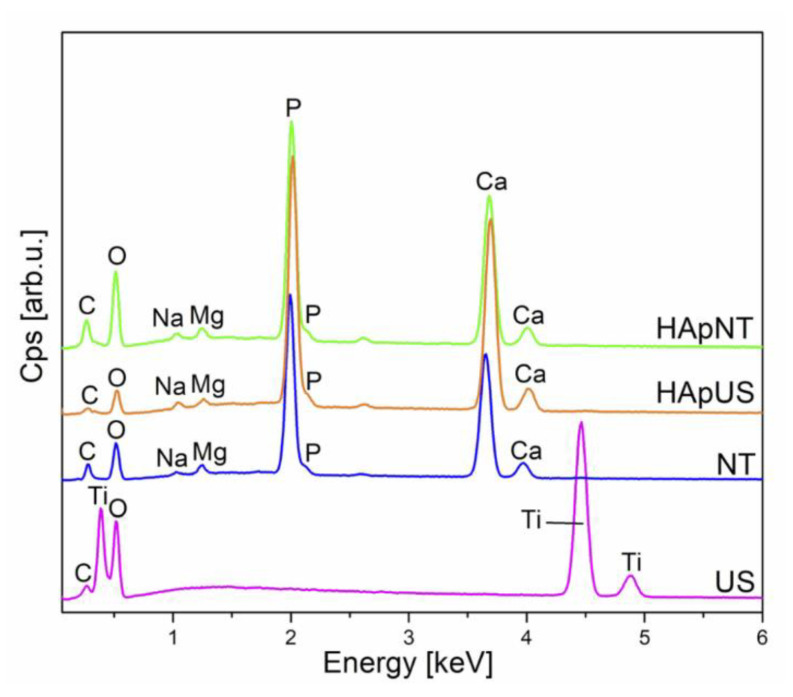
EDS spectra of NT and US samples, with and without additional HAp coating, after soaking in simulated body fluid (SBF) for 2 weeks.

**Figure 8:**
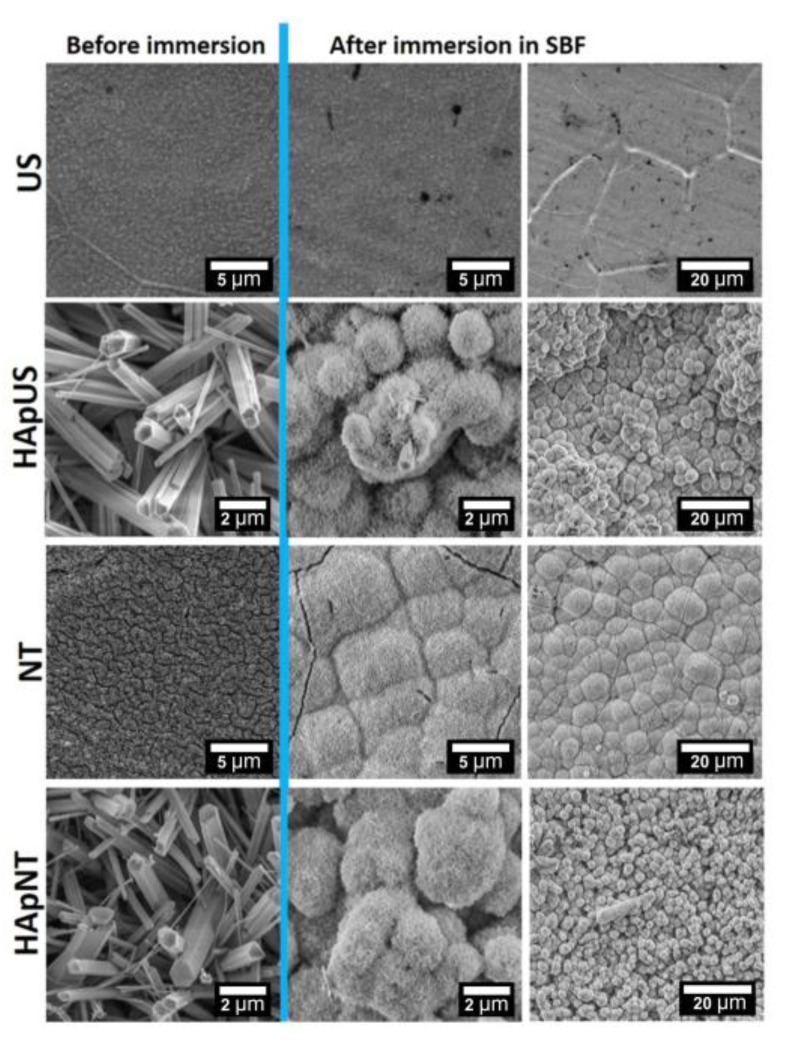
SEM images (high and low magnification) of NT and US samples, with and without additional HAp coating, performed before and after soaking in SBF for 2 weeks.

In this work, we present an EDS (Figure 7) and SEM (Figure 8) comparison study on the bioactivity of US and NT, with and without a hydrothermally synthesized HAp coating after two weeks of immersion in SBF solution. After nearly two weeks of incubation in SBF, we observed the formation of a thick layer of bone-like apatite with characteristic flower-like crystal assembly on both substrates with hydrothermally synthesized HAp coating, i.e., HApUS and HApNT (Figure 8). The main constituents that build the structure of fabricated crystalline HAp coatings are the following elements: phosphorous (P), calcium (Ca), and oxygen (O) [39]. After treatment in SBF, in addition to elements characteristic for the synthetic HAp structure, we observe in EDS spectra for HApUS and HApNT elements that are associated with a bone-like apatite structure: carbon (C), magnesium (Mg), and sodium (Na) [40]. The NT sample without HAp coating also induced the deposition of biomimetic apatite from SBF, which is in agreement with other reports on the enhancement of bioactivity in the case of crystalline TiO_2_ nanotubes [41,42]. In case of the US sample, which was not covered with HAp, there was no change in the surface morphology before and after soaking in SBF for 13 days (Figure 8), and its EDS spectrum after the bioactivity test did not show elements characteristic for the apatite structure (Figure 7). Some reports suggest that the certain crystallographic structure of titanium dioxide may affect the bioactivity of its surface in SBF [15,43]. Our XRD study (Figure 4) revealed that there is a difference in the relative intensity ratios of anatase to rutile between the US and NT substrates (the diffraction signal assigned to anatase and rutile is only attributed to titanium substrate), which might have had an influence on their bioactive behavior. On the other hand, it has been reported that the length of nanotubes could be a crucial factor in the ability to promote in vitro apatite formation in body fluids [41]. The study showed that 2 µm-long nanotubes were significantly more effective in forming bone-like apatite compared to 500 nm-short nanotubes. This observation could be another explanation why US with the height of their walls approximately ½ of their diameter were insufficient to promote bioactivity during the test. The possible mechanism for this could be attributed to small surface charge [31], insufficient mass transfer within the US structure, or surface roughness [44]. However, when US were coated with synthetic HAp under hydrothermal conditions, the US sample was competitive to NT high bioactivity after 2 weeks of incubation in SBF (Figure 8).

## 4. Discussion

From the clinical point of view, at the time of implantation, orthopedic implants are subjected to considerable mechanical stress. Most of the studies focus on the biological aspect of nanotextured implants [45,46]; however, only few scientific reports consider investigating the mechanical properties of TiO_2_ nanotubes [21,47], especially regarding resistance to scratch [48]. According to our study, nanoscale surface topography requires a more sophisticated approach than determining the adhesion strength of an NT layer only from acoustic emission examination, friction measurements, or optical microscopy assessment. A deeper insight into evaluating the resistance against scratch of nanotextured surfaces can be achieved by combining the nano-scratch test with SEM imaging [49]. During the electrochemical growth of TiO_2_ NT beneath the nanotubular layer, a barrier layer of continuous oxide film is formed at the oxide/metal substrate interface [50,51]. To date, none of the adhesion studies considered the importance of this barrier layer, thus determining the critical load at which delamination of the thorough oxide layer occurs (NT layer along with barrier layer). Using SEM imaging, it is possible to evaluate with good precision the surface damage of the nanotubular structure from normal-load lateral scratches; however, it is difficult to identify when the continuous oxide barrier layer starts to delaminate. For this reason, we performed simultaneous SEM and EDS measurements to investigate when the metallic titanium substrate started to be exposed. This allowed us to determine the critical loads necessary for a complete delamination of the titanium oxide layer (NT/US layer along with barrier layer).

## 5. Conclusions

In this study, we present a crystalline nanoporous u-shaped structure of anodized titanium with a twice higher resistance to scratch in comparison to brittle nanotubes. We consider that this substrate could be an alternative material to nanotubes and suitable as a smart drug delivery platform. Furthermore, the US titanium substrate was successfully functionalized with hydroxyapatite coating (HApUS) under hydrothermal conditions and showed high bioactivity after 2 weeks of immersion in SBF. We reported in our previous studies [30,33] the positive effect of hydrothermally synthesized HAp coatings on titanium substrates on osteoblast-like and preosteoblast cell proliferation and adhesion. Taking into consideration the combination of superior mechanical performance of the US substrate and high bioactivity of HAp coating, we are convinced, that this surface modification may improve the performance of currently used titanium-based implants for orthopedic applications.
